# Life and Mind on the Basis of Modern Medicine

**Published:** 1881-01

**Authors:** 


LIFE AND MIND ON THE BASIS OF MODERN MEDICINE.—The editor of the *Lancet* makes this comment in criticizing a letter upon the above subject: "We maintain that the non-existence of a something beyond or behind matter *cannot*, with our present knowledge, *be proved.* The statement, that function or motor force is imminent in matter is not sufficient in these days of vigorous analysis, as an explanation of the meaning of energy in organized bodies present in the state called life, and absent in death. Co-ordinated or associated action surely has a higher or deeper signification than can be predicted of corpuscular *vis insita.* This can never account for the intelligent co-operation of atoms in working out harmoniously the well-being of the individual organism."

The foregoing extract was taken from a recent number of the *Record*, and is of such importance that we place it in our editorial department. It would be well for science, for morality and for mankind, if all editors, and others, in responsible positions in the profession, had the intelligence and moral courage to appreciate these facts in a proper manner, and to give utterance to their honest convictions upon this most important subject. And now that the *Lancet* has spoken, it is sincerely hoped that lesser lights in the profession will take courage and speak "for God, for man, for duty," and thereby aid in steming the torrents of materialism and unbelief, which are sweeping so many into the great ocean of atheism! It is no part of our duty to write sermons on the inspiration of the scriptures, nor to expatiate on the evidences of Christianity; but it is our duty, as well as our privilege to *endorse* such *noble* and *truthful* utterances as stand at the head of this paragraph. Did the class who attempt to undermine religious belief, and subvert the teaching of Revelation, supply us with *any* better thing than the declarations of the lowly Nazarine; or something which could give a dying man *equal*

comfort and hope in his last hours; we might not dwell for a moment upon the infidel teaching and tendency of the present time! But, unfortunately, they offer their proselytes and followers nothing but annihilation and darkness, as the reward for their disbelief of the Christian faith! Our great surprise is that any human being possessed of sufficient intelligence to comprehend the simplest principles of logic, to say nothing of the "longing after immortality," can in the face of nature deny the eternity of the soul and a future beyond the grave!

